# Evaluating the drivers of and obstacles to the willingness to use cognitive enhancement drugs: the influence of drug characteristics, social environment, and personal characteristics

**DOI:** 10.1186/1747-597X-9-8

**Published:** 2014-02-01

**Authors:** Sebastian Sattler, Guido Mehlkop, Peter Graeff, Carsten Sauer

**Affiliations:** 1 Institute for Sociology and Social Psychology, University of Cologne, Greinstrasse 2, 50939 Cologne, Germany; 2 Cologne Graduate School in Management, Economics and Social Sciences, Albertus-Magnus-Platz, 50932 Cologne, Germany; 3 Faculty of Economics, Law and Social Sciences, University of Erfurt, Nordhaueser Strasse 63, 99089 Erfurt, Germany; 4 Institute of Social Science, Christian-Albrechts University Kiel, Westring 400, 24118 Kiel, Germany; 5 Collaborative Research Center 882, Bielefeld University, Universitaetsstrasse 25, 33615 Bielefeld, Germany

**Keywords:** Pharmaceutical cognitive enhancement, Substance abuse, Social norms, Social network, Cognitive test anxiety, Academic procrastination, Risk attitudes

## Abstract

**Background:**

The use of cognitive enhancement (CE) by means of pharmaceutical agents has been the subject of intense debate both among scientists and in the media. This study investigates several drivers of and obstacles to the willingness to use prescription drugs non-medically for augmenting brain capacity.

**Methods:**

We conducted a web-based study among 2,877 students from randomly selected disciplines at German universities. Using a factorial survey, respondents expressed their willingness to take various hypothetical CE-drugs; the drugs were described by five experimentally varied characteristics and the social environment by three varied characteristics. Personal characteristics and demographic controls were also measured.

**Results:**

We found that 65.3% of the respondents staunchly refused to use CE-drugs. The results of a multivariate negative binomial regression indicated that respondents’ willingness to use CE-drugs increased if the potential drugs promised a significant augmentation of mental capacity and a high probability of achieving this augmentation. Willingness decreased when there was a high probability of side effects and a high price. Prevalent CE-drug use among peers increased willingness, whereas a social environment that strongly disapproved of these drugs decreased it. Regarding the respondents’ characteristics, pronounced academic procrastination, high cognitive test anxiety, low intrinsic motivation, low internalization of social norms against CE-drug use, and past experiences with CE-drugs increased willingness. The potential severity of side effects, social recommendations about using CE-drugs, risk preferences, and competencies had no measured effects upon willingness.

**Conclusions:**

These findings contribute to understanding factors that influence the willingness to use CE-drugs. They support the assumption of instrumental drug use and may contribute to the development of prevention, policy, and educational strategies.

## Background

Researchers describe the attempt by healthy individuals to augment their cognitive capacities (e.g. increasing concentration, alertness, or memory) with prescription drugs – also known as cognitive enhancement (CE) – as a continuing social trend. These studies discuss the use of different drugs and drug classes as potential enhancers, such as stimulants (e.g., methylphenidate, amphetamines, or modafinil), antidementives (e.g., memantin, piracetam, or donepezil) and antidepressants (e.g., citalopram, fluoxetine or, sertraline) [1-5]. Such drugs are usually prescribed to treat diseases including narcolepsy, shift work sleep disorder, attention deficit hyperactivity disorder, dementia, Alzheimer’s disease, depression, anxiety disorders, and so on.

Research on attitudes towards, prevalence of, and motives for using cognitive enhancers has often focused on students (cf. [6]). Their temptation to use such drugs is presumed to be high because mental capacity is essential for academic success and future career opportunities. North American studies report lifetime prevalence rates of prescription stimulant use for CE as ranging from 3 to 11% [7]. Studies of German students showed a lifetime prevalence rate of 0.8% for prescription stimulant use for CE [8] and of 4.5% for multiple types of prescription medication used for CE [9]. This implies that a significant number of students are already exposing themselves to the risk of side effects and long-term health consequences. These risks include headaches, addiction, insomnia, fatal arrhythmias, excitotoxicity, reduced appetite, hypertension, anxiety, jitteriness, and personality changes (e.g. [10-14]). Not all potential side effects and negative health consequences of CE-drug use are presently known and there are additional risks of drug-drug interactions, overdose, and the use of impure substances [15-18].

While a body of literature exists, for example, on the general, non-medical (mis-)use of different types of prescription medications (including motivations such as losing weight, enhancing performance, getting high, improving mood, etc.) [17,19-26], researchers have only recently begun to direct their attention to the drivers of and obstacles to the decision to use prescription drugs specifically for the enhancement of cognitive performance (e.g., [6,8,9,27,28]). Our research has been informed by and has built upon the first body of literature. But one significant limitation of these previous studies is that they present solely correlations between socio-demographics and the use of (or willingness to use) CE-drugs, with few theory-driven explanations. Consequently, behavioral patterns, motives, and variables that influence the willingness to use CE-drugs still remain to be identified (cf. [25-27,29]).

The theoretical basis of our research rests upon sociological and economic decision-making theories [30,31] that propose that individuals (a) want to attain certain goals such as academic success, but (b) they also have beliefs about the possibilities for or restrictions to achieving these goals. These possibilities and restrictions include the characteristics of the drugs, personal characteristics, and influences from the social environment. Based on their evaluation of the situation, individuals (c) choose a behavior that best fits their individual preferences, perceived opportunities, and constraints. Consequently, we assume that individuals instrumentally decide whether or not to use drugs (e.g., [9,27,32]). Recent research found evidence for this assumption of instrumental CE-drug use, as individuals use such drugs as means to achieve certain goals (e.g., [9,33]).

### Drug characteristics

It can be assumed that individuals consider the characteristics of CE-drugs before choosing to take them or not. On the one hand, individuals might ponder the degree and probability of enhancement in order to determine whether the drugs would satisfy their needs. On the other hand, they may be unwilling to take risks that are too great and therefore also take the severity and probability of side effects into consideration. Different individuals might be willing to pay different amounts. The influence of such drug characteristics upon respondents’ willingness to take them has not been sufficiently investigated or replicated. For example, in a study by Castaldi et al. [6], risks and benefits were measured with a single question, therefore the various individual influences of these characteristics could not be determined. For health campaigns, it might be useful to investigate the degree to which potential users take health risks into consideration (cf. [6]) and are affected by potentially exaggerated benefits [33].

One study found that willingness to use a fictitious CE-drug increased in proportion to higher enhancement effect [33]. Similar results have been found for the non-medical use of prescription stimulants [19,21]. This result supports the assumption of an instrumental use of CE-drugs [9,32,33]. The effect of increased cognitive abilities on the willingness to use CE-drugs has rarely been investigated, however. Sattler et al. [33] showed that a higher probability of enhancement effects increased respondents’ willingness to use a fictitious drug. This probability reflects the fact that the impact of CE-drugs is dependent on factors such as the physical condition of individuals or their responsiveness to medical treatment [11,12,34]. We propose the following hypothesis (*H*):

*H*_*Enhancement effect (magnitude)*_*:* The higher the magnitude of enhancement effect, the higher the willingness to use CE-drugs.

*H*_*Enhancement effect (probability)*_*:* The higher the probability of enhancement, the higher the willingness to use CE-drugs.

Recent studies show that the severity and the probability of side effects serve as obstacles to drug use (e.g., [9,19,23,28,33,35]). CE-drugs can harm individuals by means of potential side effects and health risks. Avoiding such harm and refraining from drugs with more severe and likely side effects is also in keeping with our presumption of instrumental CE-drug use. Thus, we expect the following:

*H*_*Side effects (severity)*_*:* The higher the severity of side effects, the lower the willingness to use CE-drugs.

*H*_*Side effects (probability)*_*:* The higher the probability of side effects, the lower the willingness to use CE-drugs.

Some evidence concerning the effect of price has been obtained from students who stated that price is in fact a condition for using licit/illicit stimulants for purposes of CE and that they would be more willing to use them if they were inexpensive [35]. While research on illicit drugs has taken into account the elastic effect of price upon demand [36,37], to our knowledge the effects of price on demand for CE-drugs remains unknown. We assume that individuals also take the price of CE-drugs into account when deciding for or against using such drugs:

*H*_*Drug price*_*:* The higher the price, the lower the willingness to use CE-drugs.

### Social environment

Prior studies of illicit drug use [38,39] and the non-medical use of prescription stimulants such as amphetamines and methylphenidates [19,20] have demonstrated that social contexts affect the decision to consume such drugs. Social context may also affect the willingness to use CE-drugs in several ways. Potential social pressure may motivate CE-drug use, resulting in contagion effects. Learning effects can also influence decisions, as for example in the transmission of information about the pros and cons of CE-drug intake. Furthermore, social control can affect the willingness to use CE-drugs, in the sense that when others become aware of an individual’s use of CE-drugs they may punish the user with punitive behavior such as social disapproval.

#### Peer prevalence

CE-drug use by others has been discussed as an inducement to their use [2,40]. Individuals who refrain from using them may have relative disadvantages [28,40] because they may need to work harder to keep up with users. A recent study [28] also found the same contagion effect in the case of a fictitious CE-drug. However, Franke et al. [35] found that the majority of respondents (66%) would (very) likely refrain from using licit/illicit stimulants for purposes of CE even if others used them. Prevalent use of drugs may also be an indication of these drugs having a good risk-benefit ratio [28] and/or being morally acceptable (cf. [41,42]). Thus, we expect the following:

*H*_*Peer prevalence*_*:* The higher the peer prevalence, the higher the willingness to use CE-drugs.

#### Social suggestions

Research suggests that advice from the social network stimulates CE-drug use [6], while Franke et al. [35] found that only a few respondents stated that they would think about using licit/illicit stimulants for CE if their employers recommended them. We expect the following:

*H*_*Social suggestions*_*:* The more positive advice received concerning CE-drug use, the higher the willingness to use CE-drugs.

#### Social disapproval

Social control in terms of social disapproval (cf. [43-45]) might present an obstacle to the willingness to use CE-drugs. Strong disapproval by others may lower willingness to use CE-drugs. Some studies have been able to confirm this hypothesis for the non-medical use of prescription stimulants [19,20]. Social disapproval indicates that CE conflicts with social/group norms. Violating such norms may lead to costs in the form of informal punishment [44,45], such as exclusion from the group. Thus, we expect the following:

*H*_*Social disapproval*_*:* The higher the level of social disapproval, the lower the willingness to use CE-drugs.

### Personal characteristics

Personal characteristics, such as a lack of competencies (e.g., [24-26]) or motivation, the tendency to procrastinate, and the experience of cognitive test anxiety (CTA) [9], may be factors in explaining the willingness to use CE-drugs because they may produce a demand for pharmaceutical agents. These characteristics may hamper academic performance, (e.g., [46-49]), and CE-agents may help students to cope with related (subjectively perceived) deficits and their negative consequences. Similar assumptions can be derived from the Strain Theory, which has been used to explain the non-medical use of prescription stimulants [22]. Most of these variables have not been investigated in terms of the willingness to use CE-drugs.

The question has been raised of whether CE-drug users aim to enhance their performance above average or to achieve an average level of performance [50]. It can be argued that students with low self-efficacy see themselves as less skilled or less successful [51] and attempt to attain success by other means. Consequently, they may have a higher incentive to use CE-drugs to compensate for their perceived lack of competence and to catch up to their peers (cf. [9,25]). Several studies (e.g., [19,24,26]) have found that lower grades and lower self-assessed competencies were associated with increased non-medical use of stimulants. Consequently, we expect the following:

*H*_*Competencies*_*:* The higher the self-assessed competencies, the lower the willingness to use CE-drugs.

The tendency to take risks may be influential because CE-drug use has potential negative effects on health and is therefore a risky behavior. Prior research has shown that risk-averse individuals are more likely to refrain from CE-drug use [9]. Therefore, we expect the following:

*H*_*Risk attitudes*_*:* The higher the tendency to take risks, the higher the willingness to use CE-drugs.

Students might instrumentally use drugs because of their ‘subjectively attributed or real beneficial effects’ in coping with CTA and avoiding its negative consequences (cf. [9,32]). These consequences occur because CTA negatively affects test preparation and test-taking due to impaired working memory [49,52]. Although no effect of CTA was found in a small-scale investigation using a convenience sample of students from a German vocational school [27], a recent study [9] found that higher levels of CTA increased the frequency of CE-drug use. We expect the following:

*H*_*Cognitive Test Anxiety*_*:* The higher the level of CTA, the higher the willingness to use CE-drugs.

#### Study motivation

For intrinsically motivated students, academic achievement and learning are ends in themselves, and these students achieve satisfaction from mastering a task (e.g., [53-56]). Taking CE-drugs might undermine their sense of personal achievement and reduce their satisfaction (cf. [57]). Empirical studies reveal that intrinsically motivated students are less competitive and less focused on good grades (e.g., [56,58]), and thus that gaining competitive advantage over fellow students by taking CE-drugs might be less important. Therefore, intrinsic motivation is expected to be a protective factor against willingness to use CE-drugs:

*H*_*Study motivation*_*:* The more students are intrinsically motivated in their studies, the lower their willingness to use CE-drugs.

#### Academic procrastination

Like CTA, academic procrastination results in time pressure and decreased academic performance [46,47]. This pressure can be seen as a crucial source of strain [22]. Some students attempt to cope with this strain by cheating or plagiarizing [59], whereas for other students, it can be assumed that CE-drug use is an attractive coping strategy:

*H*_*Academic procrastination*_*:* The more often students procrastinate, the higher their willingness to use CE-drugs.

CE-drug use is also connected to social norms such as fairness and authenticity [2,60,61]. While social norms have been found to be strong predictors of the consumption of illicit drugs such as marijuana [62,63] and the non-medical use of prescription stimulants [19], they have rarely been investigated in studies focusing specifically on CE-drugs [33], which is another research gap [60]. Violating internalized social norms against CE-drug use might result in internal penalties (i.e. psychological costs) and we expect this to impede the willingness to use CE-drugs (cf. [33,43,64]). Thus, we expect the following:

*H*_*Internalized social norms*_*:* The more strongly norms against CE-drug use are internalized, the lower the willingness to use CE-drugs.

Previous studies [9,19,23] have shown that prior drug use increased the likelihood of prescription drug use for non-medical reasons including CE. This effect has been interpreted as a result of preferences related to previous decisions and conditions [9,65,66]. This finding may also reflect the fact that students are likely to repeat behavior that was ‘experienced as either successful or less burdensome’ after passing habituation processes [9,32,67]. Personal experiences with the non-medical use of prescription drugs have been found to reduce perceived side effects, increase perceived benefits, and increase subsequent use, (e.g., [23,24,68]). Consequently, we expect the following:

*H*_*Prior CE-drug use*_*:* Prior CE-drug use increases willingness to use CE-drugs.

**Figure 1:**
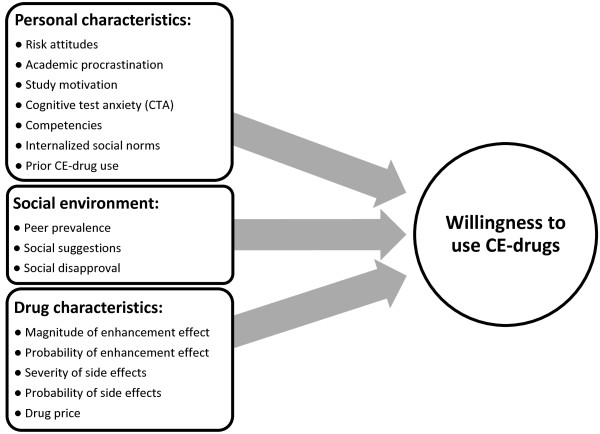
Factors influencing the willingness to use CE-drugs.

It is essential to obtain insight into the factors that influence the willingness to use CE-drugs in order to develop prevention, policy, and educational strategies [25]. Such strategies may be needed due to the potential social problems related to CE-drug use, such as (a) the social pressure of abstainers to take CE-drugs to keep up with users, (b) the harmful side effects for users, and (c) societal burden upon the health care system. While the current prevalence of CE-drug use is relatively low (see above), several researchers have forecasted an increased consumption of such substances (e.g., [3,6,69,70]). Therefore, besides only monitoring the development of prevalence rates, it is important to investigate factors influencing the potential use of such drugs. Previous research on the (illicit) use of tobacco, amphetamines, and marijuana, for example, has shown that willingness measures can be used to predict subsequent behavior (e.g., [71,72]). The use of a willingness measure allowed us to experimentally vary and test the influence of several hypothetical characteristics of CE-drugs and of the social context upon the decision-making process. We were also able to investigate potential future scenarios and their impact on the respondents’ willingness to use CE-drugs, such as a significant amount of performance enhancement with or without side effects. To sum up, our study aims to investigate the influence of personal and contextual factors as well as drug characteristics (see hypotheses below) on students’ willingness to use CE prescription medication without any medical necessity (see Figure 1). Thus, we address some limitations of prior studies (such as small-scale convenience samples or a focus on limited sets of factors).

## Methods

### Participants and survey method

In January 2011, we conducted a self-administered, fully anonymous web survey during the biannual longitudinal FAIRUSE survey on study conditions and academic cheating (cf. [9])^a^. Four German universities were randomly selected for the study. Within these universities, we randomly selected 175 students from 14 randomly drawn academic disciplines with n_students_ > 175. Furthermore, we sampled 300 students from all other disciplines with n_students_ < 175. Our survey was sent initially to a total of 11,000 students (2,750 per university) from 138 disciplines.

The second wave of this study – which is the basis for this paper – invited the participation of students who a) had completed the survey in the first wave, b) did not finish their studies, c) did not change their university, or d) dropped out of their university. We therefore sent pre-notification letters by mail to 5,048 students who met these criteria; the letters explained the study’s purpose and data protection strategy and included a protection declaration. Approximately one week later, we emailed invitations and personal access codes for the survey followed by up to two reminder emails. At the end of the survey, students could choose a 5 Euro incentive in the form of money sent by mail or via PayPal, vouchers for an online retailer, or donations to UNICEF or Amnesty International.

Our response rate from the second wave of 69.1% (n = 3,486) is similar to the rates of other studies in this field [24,73,74]. Complete responses for all analyzed variables were available for 2,877 respondents. To test whether missing data caused by item non-responses and dropout influenced our results, we used a multiple imputation procedure by chained equations [75]. Because results did not change significantly (results available upon request), listwise deletion was used. Two out of five (39.3%) participants were male (see Table 1 for all demographic information). Age was measured in intervals of two years to provide more privacy. The median age was 22–23 years. Students from mathematics or natural sciences represented 39.2% of the respondents.

**Table 1:** Descriptive statistics for the demographic variables

**Variable**	***Total***	***Percent of this sample***	***Percent of the population***
Gender			
*▪ Female*	1,753	60.7	53.5
*▪ Male*	1,134	39.3	46.5
Age			
*▪ <18*	1	0.0	0.0
*▪ 18-19*	37	1.3	4.3
*▪ 20-21*	744	25.8	21.3
*▪ 22-23*	864	29.9	23.8
*▪ 24-25*	646	22.4	20.8
*▪ 26-27*	282	9.8	12.6
*▪ 28-29*	143	5.0	6.9
*▪ 30-31*	68	2.4	3.6
*▪ 32-33*	32	1.1	2.0
*▪ 34-35*	24	0.8	1.2
*▪ 36-37*	7	0.2	0.7
*▪ 38-39*	7	0.2	0.6
*▪ 40-41*	7	0.2	0.5
*▪ >41*	25	0.9	1.5
Field of study			
*▪ Sports*	92	3.2	3.5
*▪ Linguistic and cultural studies*	943	32.7	25.9
*▪ Legal, economic, and social sciences*	309	10.7	31.6
*▪ Mathematics and natural sciences*	1,135	39.3	23.3
*▪ Human medicine and health*	70	2,4	6.5
*▪ Agriculture, forestry, and nutritional sciences*	85	2.9	1.0
*▪ Engineering*	165	5.7	6.5
*▪ Arts and science of art*	88	3.1	1.8

### Ethics statement

Our research was guided by the principles formulated in the WMA Declaration of Helsinki. No ethics approval is needed for social science research in Germany as long as it does not refer to matters regulated by law, such as the German Medicine Act (AMG), the Medical Devices Act (MGP), the Stem Cell Research Act (StFG), or the Association’s Professional Codes of Conduct. Therefore, no approval was needed for our study. According to paragraph 28 of the Data Protection Act of North Rhine Westphalia, we used a fully anonymous research design. Several means have been employed to ensure the voluntariness, confidentiality, and anonymity of our survey, which were emphasized in all communications with respondents: The partnering universities never had access to response data, and the researchers never had access to any of the respondents’ personal data. Furthermore, secure sockets layer (SSL) protocols were used to protect answers while responding. Participants were informed about the anonymity and purpose of the survey via postal letters, in all subsequent e-mails, and on the first survey page. Therefore, participation can be understood as a conclusive action. All our procedures and data collection were approved by the legal services of Bielefeld University and supervised by an official data protection officer.

### Predictors assessed using a factorial survey design

We applied a factorial survey [76,77] in which respondents evaluated descriptions (so-called vignettes) of a person facing the decision of whether or not to take a hypothetical CE-drug (cf. [28,33], see the discussion section for limitations of this approach). We experimentally varied five characteristics (dimensions) of this drug (magnitude, probability of enhancement effect, severity, probability of side effects, and drug price), and three characteristics of the social environment (peer prevalence, social disapproval, and social suggestions; see Table 2). Each of these eight dimensions consists of three levels, implying a total of (3^8^ = 6,561 possible combinations, of which 600 were chosen via D-efficient sampling, (e.g., [78]). D-efficient designs are constructed by using an algorithm specifying a sample characterized by minimal correlation between the dimensions and, at the same time, maximal variance and balance of the frequency of the vignette levels. The sampling procedure was done with SAS software. Each respondent was randomly assigned to one of the 600 vignettes. Each single vignette was rated multiple times by different respondents^b^. After reading the vignette, the respondents were asked the following question: ‘Would you consume the drug if you were in her position?’ They rated their willingness to use the drug on a 10-point scale ranging from ‘strongly against use’ (0) to ‘strongly in favor of use’ (9).

**Table 2:** Descriptive statistics for the independent metric variables measuring personal characteristics

**Variable**	***Response options***		***Min***	***Max***	***Mean***	***Standard deviation***
	***Left anchor***	***Right anchor***				
Risk attitudes	Not at all willing to take risks	Very much willing to take risks	1	11	5.47	2.283
Academic procrastination	Very seldom	Very often	1	6	2.77	0.978
Study motivation	Do not agree at all	I agree completely	1	6	4.68	1.074
Cognitive test anxiety (CTA)	Not true at all	Completely true	1	4	3.07	0.718
Competencies	Very difficult	Very easy	1	5	3.27	0.585
Internalized social norms	Absolutely moral	Absolutely not moral	1	7	5.58	1.714

### Predictors assessed with the survey

#### Risk attitudes

Respondents were asked the following question: ‘Are you generally a person who is fully prepared to take risks, or do you try to avoid taking risks?’ They rated their risk attitudes on an 11-point scale ranging from ‘not at all willing to take risks’ (1) to ‘very much willing to take risks’ (11). This measure has been experimentally validated and showed high stability in prior research [79].

#### Cognitive test anxiety (CTA)

The cognitive dimension of the German version of the Test Anxiety Inventory was used to assess CTA [80]. We selected five items (e.g., ‘I am thinking about the consequences of failing’) based on factor loadings in previous studies [81]. We used a four-point scale ranging from ‘not true at all’ (1) to ‘completely true’ (4). The internal consistency (α = 0.87) was acceptable compared to the original scale (α = 0.91; [82]).

#### Academic procrastination

We used the Questionnaire for Academic Procrastination (QAP) [83]. Conceptually, this instrument refers to the intention-action gap, or the problem of not turning intentions into the desired actions (cf. [84,85]). It covers different stages of task processing. The frequency of eight behaviors (e.g., ‘Even if I intend to finish a university assignment, I do not do it.’) was rated on a six-point scale ranging from ‘very seldom’ (1) to ‘very often’ (6). The internal consistency was good (α = 0.93).

#### Competencies

Students rated their academic skills using six items (e.g., *‘*handling a typical question in their subject’) from the ‘Evaluation in Higher Education: Self-Assessed Competences’ instrument (HEsaCom; [86]) on a five-point scale from ‘very difficult’ (1) to ‘very easy’ (5). The alpha score (0.78) of this scale was acceptable but slightly below the original scale. Students were also asked for their grades. Due to a higher number of missing values in this measure and similar effects on the willingness to use CE-drugs (results not discussed here), we used the self-rated competencies measure only.

#### Study motivation

Intrinsic study motivation was assessed by asking the question, ‘Why do you learn and study in your main subject?’ (cf. [87,88]). Three items, such as ‘I learn and work because the study content corresponds to my personal preferences’, were rated on a six-point scale ranging from ‘do not agree at all’ (1) to ‘I agree completely’ (6). The scale had an internal consistency of α = 0.91.

#### Internalized social norms

Respondents disclosed their internalization of social norms concerning CE-drug use by answering the question [33], ‘How do you personally evaluate the use of prescription drugs to enhance work performance without any medical necessity? I think the use is…’ and three items: ‘before an examination’, ‘during an examination’, and ‘in general for university studies’. Responses were rated on a seven-point scale ranging from ‘absolutely moral’ (1) to ‘absolutely not moral’ (7). Our measure revealed a high internal consistency (α = 0.94).

#### Prior CE-drug use

Prior use was assessed with the question, ‘There are students who enhance their cognitive efficiency using prescription medicine without any medical necessity. Have you ever done that?’ and five response options: ‘never’ (0), ‘last 30 days’ (1), ‘last 30 days to 6 months’ (2), ‘6 months to 1 year’ (3), and ‘>1 year’ (4) (cf. [9]). To distinguish non-users from users and because of the low prevalence, we deployed a binary coding (portion of non-users = 97.2%; users = 2.8%). This prevalence rate of prescription drug use for CE is low but nonetheless still within the range of prevalence rates found in prior German studies [8,27,40]. The results, however, are not entirely comparable, due to the different sampling strategies used and different definitions of CE-drugs, for example. [5,89].

### Statistical analysis

Within the factorial survey, almost two-thirds (65.3%) of the respondents strongly refused to take the presented CE-drug, whereas the others were more willing to consume the drug. Due to overdispersion in the data (*Mean* = 1.08; *SD* = 2.019), we applied a multivariate negative binomial regression model that produces more efficient and less-biased estimates than ordinary least squares models [90] or Poisson models. Wald tests were applied to assess the statistical significance of the coefficients presented in the results section.

## Results

### Drug characteristics

Table 3 shows that the respondents’ willingness to take the drug increased by a factor of 1.329 or 32.9% (z = 3.25; p < 0.001) when the described drugs tripled the amount of memorized information compared to a 5% increase. A doubled amount had no effect (z = 0.11; p = 0.914). An enhancement effect occurring with a probability of 50% (z = 2.83; p = 0.005) or 100% (z = 3.86; p < 0.001) significantly increased the willingness compared to a probability of 5%. The severity of side effects had no significant effect. However, when every user had to fear side effects compared to only one of 1,000,000 users, respondents were less willing to use the drug (z = -5.16; p < 0.001). Compared to this latter category, no deterrent effect of side effects occurred in one of 1,000 users (z = -0.57; p = 0.567). A price of 100 Euros for 10 pills also reduced the willingness to use a pill compared to free pills (z = -4.22; p < 0.001).

**Table 3:** Vignette dimensions and levels used in this study: experimental variation of five drug characteristics and three characteristics of the social environment

**Dimension**	**Levels**
Peer prevalence	A student considers using a prescription drug to enhance her memorization skills for her exam preparation. From a medical point of view, this is not necessary. This student knows that
	*▪ none*
	*▪ every second*
	*▪ every one*
	of her friends or acquaintances uses such substances.
Social suggestions	She
	*▪ never*
	*▪ sometimes*
	*▪ very often*
	gets suggestions from others to try such means.
Magnitude of enhancement effect	By taking such drugs, she hopes to increase the amount of memorized information by
	*▪ 5 percent*
	*▪ a factor of two*
	*▪ a factor of three*
	compared to her normal state.
Probability of enhancement effect	From a recently published study, she knows that that the effect occurs with a
	*▪ 5*
	*▪ 50*
	*▪ 100*
	percent chance.
Probability of side effects	This study also reported that
	*▪ one of 1,000,000 users*
	*▪ one of 1,000 users*
	*▪ every user*
Severity of side effects	developed
	*▪ very light*
	*▪ moderate*
	*▪ very strong*
	depression. Further side effects are unknown.
Drug price	Someone can provide her with a package of 10 pills for
	*▪ free.*
	*▪ 20 Euros.*
	*▪ 100 Euros.*
	This is enough for 20 learning hours.
Social disapproval	The use of such drugs would cause
	*▪ no*
	*▪ moderate*
	*▪ very strong*
	criticism in her environment

### Social environment

We found greater willingness to use the drug when every second peer (z = 2.95; p = 0.003) or every peer used it (z = 3.19; p = 0.001) compared to a situation in which no friends or acquaintances used such drugs. Social suggestions had no effect, but students were deterred from usage when they received very strong social criticism regarding usage compared to no criticism (z = -3.87; p < 0.001).

### Personal characteristics

We found that risk attitudes (z = 0.72; p = 0.470) and competencies (z = 0.67; p = 0.501) had no influence on the responses. Individuals who tended to procrastinate (z = 2.86; p = 0.004), less intrinsically motivated individuals (z = -3.15; p = 0.002), and students with higher CTA scores (z = 6.05; p < 0.001) were more willing to use CE-drugs. Higher levels of norm internalization against CE-drug use reduced the willingness to use such drugs (z = -17.85; p < 0.001). Respondents who had already used CE-drugs were more willing to use a drug compared to students who had never used such drugs (z = 6.85; p < 0.001).

### Demographic controls

Gender (z = -0.65; p = 0.515) and age (z = -1.42; p = 0.156) did not affect the respondents’ willingness to use a CE-drug. We found the lowest willingness among sports students compared to engineering students (z = 2.28; p = 0.023).

## Discussion

By analyzing the influence of distinct types of potential factors explaining the willingness to use CE-drugs, this study contributes in multiple ways to an understanding of the drivers and obstacles related to CE-drug use.

### Drug characteristics

As previously found [33] and as assumed in our hypothesis (*H*_*Enhancement effect (magnitude)*_), respondents were more willing to use CE-drugs when their enhancing effect was very strong. However, to date, the magnitude of the performance enhancement of available CE-drugs is small to moderate for healthy users [14]. A tripled amount of memorable information might be not achievable today, but testing this potential future scenario reveals interesting insights about future trends or exaggerated expectations. Similar to another study [33] and in line with *H*_*Enhancement effect (probability)*_, the probability of enhancement effects increased respondents’ willingness to use these drugs. The effects found here indicate that individuals consider the magnitude and probability of enhancement when facing the decision to use CE-drugs, which supports the assumption of an instrumental CE-drug use [9,32,33].

Previous research, (e.g., [9,23]), has found that more significant side effects are associated with a lower willingness to engage in non-medical drug use. In studies similar to ours, one study found that side effects such as increased severity of headaches [33] reduced the willingness to use a fictitious CE-drug. A second study found that the possibility of more severe side effects decreased the respondents’ willingness to use drugs compared to the possibility of only mild side effects [28]. In this study, we could not replicate this deterrent effect. Therefore, *H*_*Side effects (severity)*_ has to be rejected in our study. The reasons for this should be investigated in future research. However, in line with the instrumental use of CE-drugs and with *H*_*Side effects (probability)*_, a high likelihood of side effects decreases the willingness to use CE-drugs. This finding conforms to prior research [28,33,35].

As hypothesized (*H*_*Drug price*_), we found that a high price decreased the willingness to use CE-drugs. This finding is in line with economic studies on the supply and demand of illicit drugs (see for instance [36,37]) and confirms the finding from another CE study [35] that the price is important.

### Social environment

According to *H*_*Peer prevalence*_, our results show greater willingness when more peers use CE-drugs. Another study has also provided evidence of such a contagion effect [28]. But Franke et al. [35] showed that only a minority of respondents (7.5%) would also consume licit/illicit stimulants for purposes of CE when friends did so.

Suggestions by others to use CE-drugs did not alter the respondents’ willingness to take CE-drugs, consequently *H*_*Social suggestions*_ has to be rejected. Another survey has shown that only 5.7% of the respondents would use CE-drugs if employers recommended their use. Future research can investigate whether recommendations by others than employers are (more) influential.

In line with *H*_*Social disapproval*_, the willingness to use CE-drugs was lower when others strongly disapproved of their use. This can be interpreted as a social control effect. Respondents appear to seek to avoid the cost of informal punishment by others.

### Personal characteristics

While several studies (e.g., [24,26]) have found that (self-assessed) performance measures were associated with increased non-medical use of prescription drugs, we did not find such an effect from self-assessed competencies (cf. [68] and [9] for 6-months use frequency) on the willingness to use a CE-drug. Therefore, *H*_*Competencies*_ has to be rejected for our data.

No effect was found from risk attitudes. This finding contradicts *H*_*Risk attitudes*_ and prior research that found that risk-averse individuals used CE-drugs less often [9]. Future research should clarify this ambivalence.

In line with a recent study [9] and *H*_*Cognitive Test Anxiety*_, we found that higher levels of CTA increased the willingness to use CE-drugs. This can be understood as a coping strategy to deal with CTA and its negative consequences. However, the findings of another investigation among students from a German vocational school [27] were not supportive of this hypothesis. The meaningfulness and interpretability of this investigation is limited, as it is based on a small-scale convenience sample.

We found that students with higher intrinsic motivation to study were less willing to take CE-drugs. Thus, *H*_*Study motivation*_ is confirmed by our data. Our results indicate that such motivation can be seen as a protective factor against CE-drug use, because CE-drug use might conflict with achieving satisfaction through hard work.

Our study is the first to show that academic procrastination increases the willingness to use CE-drugs. Consequently, *H*_*Academic procrastination*_ was confirmed. Our underlying assumption was that individuals try to cope with the negative consequences of procrastination by using CE-drugs.

We confirmed the result of a prior study on CE-drugs [33] by showing that a stronger internalization of social norms decreases the willingness to use a CE-drug. This finding also confirms *H*_*Internalized social norms*_. The strong effect found here implies that internalized norms are a major factor in the decision-making process about morally questionable behavior and that internal penalties occurring in the case of norm violations might be very costly to the individual.

Similar to a prior study on CE-drug use [9] and to our hypothesis (*H*_*Prior CE-drug use*_), we found that prior CE-drug use increased the willingness to use CE-drugs. This effect can have several meanings: it can be interpreted as behavior guided by habits or as a result of preferences related to previous decisions and conditions of decision-making. Future research should determine which interpretation is accurate.

Similar to several previous studies on the non-medical use of prescriptions, we found no significant gender differences (e.g., [24,25,74]) although other studies have reported higher levels of non-medical drug use among men [24,26,91]. Further research is required to clarify a) whether gender differences exist and b) what causes potential differences.

No age effects occurred in our study (cf. [26,28]) although some studies [17,92] have found that older students admitted to having used non-prescription drugs more often. An investigation of the reasons for this finding is left for future research.

Sports students revealed the lowest values regarding willingness to use CE-drugs, whereas the highest values were found among engineers. However, prior studies [8,91,93] found no clear pattern in terms of academic discipline. Therefore further investigation is needed.

### Limitations of the study

This study has several limitations. Because not all sampled students participated, potential selective non-response could bias the results. However, our response rate of 69.1% is similar to previous studies in this field [8,29,73]. We also assessed the influence of selective dropout on the study results. To do so, we calculated sampling weights with information about the proportions of each gender, age group, and academic discipline from the basic population of the four universities (see Column 4 in Table 1) and reran the regression model presented in Table 4[94]. There were no significant changes in the results, indicating that sample composition biases did not influence the interpretation of the results with respect to our hypotheses.

**Table 4:** Multivariate negative binomial regression model on the willingness to use a CE-drug (n = 2,887)

	***IRR***^***a***^	***95% CI***^***b***^
***Drug characteristics***		
Magnitude of enhancement effect (Ref. 5 percent):		
*▪ a factor of two*	1.010	[0.849,1.201]
*▪ a factor of three*	1.329^**^	[1.120,1.577]
Probability of enhancement effect (Ref. 5 percent):		
*▪ 50 percent*	1.287^**^	[1.081,1.533]
*▪ 100 percent*	1.392^***^	[1.177,1.646]
Severity of side effects (Ref. very light depression):		
*▪ moderate depression*	0.924	[0.781,1.093]
*▪ very strong depression*	0.881	[0.746,1.041]
Probability of side effects (Ref. one of 1,000,000 users):		
*▪ one of 1,000 users*	0.955	[0.815,1.119]
*▪ every user*	0.634^***^	[0.533,0.754]
Drug price (Ref. free):		
*▪ 20 Euros*	1.118	[0.955,1.310]
*▪ 100 Euros*	0.683^***^	[0.572,0.815]
***Social environment***		
Peer prevalence (Ref. none):		
*▪ every second*	1.287^***^	[1.089,1.522]
*▪ every one*	1.321^**^	[1.113,1.568]
Social suggestions (Ref. never):		
*▪ sometimes*	0.887	[0.749,1.051]
*▪ very often*	1.071	[0.907,1.265]
Social disapproval (Ref. no criticism):		
*▪ moderate criticism*	0.926	[0.788,1.090]
*▪ very strong criticism*	0.713^***^	[0.600,0.846]
***Personal characteristics***		
Risk attitudes	1.012	[0.980,1.045]
Academic procrastination	1.112^**^	[1.034,1.196]
Study motivation	0.897^**^	[0.839,0.960]
Cognitive test anxiety (CTA)	1.383^***^	[1.245,1.535]
Competencies	1.048	[0.915,1.199]
Internalized social norms	0.673^***^	[0.644,0.702]
Prior CE-drug use	2.409^***^	[1.873,3.097]
***Demographic variables***		
Male (Ref. female)	0.949	[0.812,1.110]
Age^c^	0.969	[0.928,1.012]
Field of study (Ref. sports):		
*▪ Linguistics & cultural studies*	1.260	[0.792,2.004]
*▪ Legal, economic, & social sciences*	1.237	[0.763,2.006]
*▪ Mathematics & natural sciences*	1.261	[0.797,1.995]
*▪ Human medicine & health*	1.272	[0.710,2.279]
*▪ Agriculture, forestry, & nutritional sciences*	1.285	[0.716,2.307]
*▪ Engineering*	1.845^*^	[1.089,3.126]
*▪ Arts & science of art*	1.669	[0.981,2.839]
Log-pseudolikelihood (full model)	-3566.2	
Log-pseudolikelihood (base model)	-3848.1	

*p < 0.05, **p < .01, ***p < .001 (Wald tests, robust standard errors).

a Adjusted Incidence Rate Ratios (*IRR*).

b Confidence Intervals (*CI*).

c Age was treated as a metric variable (see Table 2 for the 14 measured categories).

The question about the willingness to use CE-drugs might be seen as sensitive due to the normative dimension of CE-drug use (cf. [28]). For sensitive questions, non-anonymous surveys may result in underreporting [95,96]. As described in the methods section, our study was fully anonymous; answers were protected via SSL protocols, and an official data protection officer monitored adherence to the data protection strategy. Data security principles were emphasized in all communications with respondents. Generally, respondents in web surveys reveal higher levels of sensitive information than respondents in computer-assisted telephone interview surveys [97]. One indication of the confidentiality of the survey is that only 19 participants (0.5%) refrained from answering the question about their willingness to use CE-drugs. Moreover, due to the hypothetical nature of factorial surveys, responses are less prone to response bias than direct questioning surveys [98-100]. Finally, we tested whether perceptions of anonymity regarding the survey influenced the reported willingness, but we found no effects (results are available upon request).

Our study utilized a willingness measure to use CE-drugs. Such measures do not fully correspond to actual behavior. The conditions presented in the vignettes might differ from the factors actually influencing a respondent’s decisions. However, there is evidence from research on the (illicit) use of tobacco, amphetamines, or marijuana, for example, that willingness measures are factors that do influence behavior (e.g., [71,72]). Our approach allowed us to experimentally vary and test the influence of several hypothetical characteristics of CE-drugs and of the social context in the decision-making process. Consequently, potential and interesting future scenarios could be investigated as well such as a high magnitude of performance enhancement or a social environment very friendly to CE. Another advantage of willingness measures is their lower sensitivity. Thus, fewer refusals or distorted answers can be expected (e.g., [72]). Furthermore, when attempting to study behavior several problems need to be considered; it can, for example, be problematic to explain past behavior by means of factors measured after the occurrence of this behavior because such factors might change over time. Panel designs as well might overlook these changes [43]. However, investigating the hypotheses of our study with behavioral measures in future research is worthwhile.

Another potential limitation is that we only investigated university students in one country. Cultural differences, the legal status of drugs, and drug availabilities vary across countries and may affect the willingness to use CE-drugs (e.g., [8,9]). Therefore, future studies should replicate our study in other cultural contexts.

## Conclusion and implications

By analyzing various drivers of and obstacles to the willingness to use CE-drugs, this study aimed to increase our understanding of decisions regarding their use. As such, it provides a necessary supplement and corrective to the limitations of previous research. Not only do researchers assume that CE-drug use [6,69] will increase in the future, but also many factors influencing the use of CE-drugs have not yet been investigated (or their effects have not been replicated). Not understanding the potential causes of CE-drug use is and remains an obstacle in developing policy, intervention, and prevention. We have found that several factors increase the willingness to use CE-drugs, whereas other factors decrease it, and some have no effect. Thus, we can generally confirm our assumption that CE-drug use is an instrumental behavior in the sense of a rational choice (cf. [9,28,32]). Several factors may increase the perceived usefulness of CE-drug consumption and therefore may turn it into a preferred strategy. For example, students may expect these drugs to help them cope with certain personal deficits or restrictions in achieving academic success (cf. [9]). Their use could be associated with the anticipation of rewards or the avoidance of negative consequences, including costs such as relative disadvantages when many peers are assumed to medically augment their performance. Other constraints on the willingness to use CE-drugs instrumentally were also considered, such as the potentially deterrent effect of high prices, the high likelihood of side effects, or strong social disapproval.

Our results have several implications that can be utilized for interventions and policy regulations. For instance, willingness to use CE-drugs only increased if the magnitude of the enhancement was likely and extraordinary. However, people can be informed that such medication seems not to exist at the moment (e.g., [14]) to avoid exaggerated perceptions and to reduce the inclination to use CE-drugs. Castaldi et al. [6] suggest health campaigns to inform the public about the negative consequences of CE-drugs. This idea is supported by the deterrent effect of very probable side effects. Because internalized social norms against CE-drug use decreased the willingness to use CE-drugs, policymakers could consider means to strengthen such norms, such as defining them as misconduct and including their disaffirmation in university honor codes [cf. 33]. Furthermore, the effects of other personal characteristics can be employed to reduce risky self-medication. The willingness to engage in CE-drug use can be described as an instrumental coping strategy to address the anticipated negative effects of CTA (cf. [9]). Means to reduce CTA (e.g., behavioral or cognitive-focused interventions) or its negative consequences (e.g., social support) could decrease the benefits of CE-drug use (cf. [101,102]). Because the willingness to take CE-drugs decreases with increasing intrinsic study motivation, a deterrent to drug use could involve fostering mastery goal orientation among students, for example by increasing autonomy in selecting learning contents or encouraging students to take intellectual risks instead of penalizing mistakes (cf. [55]). Further research should investigate the influence of the suggested means of regulating CE-drug use.

## Endnotes

^a^Two variables (risk attitudes and intrinsic study motivation) were assessed in wave 1 of this biannual study, and all the others were measured in wave 2. For the sake of brevity, we only describe details for the second wave here (see [9] for details about wave 1).

^b^Within our design, we also investigate rare cases or hypothetical (future) scenarios to explore their theoretically interesting effects (e.g., drugs tripling the amount of memorized information but causing very strong depression in every user). None of these is illogical or unimaginable. The very low numbers of students who refused to answer the vignette (n = 18) or dropped out on this page (n = 1) can be seen as indicators that our vignettes were easy to answer.

## Competing interests

The authors declare that they have no competing interests. The authors did not receive any research support from public or private actors in the pharmaceutical sector.

## Authors' contributions

SS and CS designed the study. SS conducted the field works. SS and CS performed the statistical analysis. SS, GM, PG, and CS participated in writing the manuscript. All authors read and approved the final manuscript.
